# Influence of Obesity and Type 2 Diabetes on Calcium Handling by Skeletal Muscle: Spotlight on the Sarcoplasmic Reticulum and Mitochondria

**DOI:** 10.3389/fphys.2021.758316

**Published:** 2021-11-02

**Authors:** Hiroaki Eshima

**Affiliations:** Department of International Tourism, Nagasaki International University, Nagasaki, Japan

**Keywords:** sarcoplasmic reticulum, mitochondria, calcium, obesity, diabetes, skeletal muscle

## Abstract

Obesity and diabetes have been shown to interfere with energy metabolism and cause peripheral insulin resistance in skeletal muscle. However, recent studies have focused on the effect metabolic insult has on the loss of muscle size, strength, and physical function. Contractile dysfunction has been linked to impaired intracellular Ca^2+^ concentration ([Ca^2+^]_i_) regulation. In skeletal muscle, [Ca^2+^]_i_ homeostasis is highly regulated by Ca^2+^ transport across the sarcolemma/plasma membrane, the golgi apparatus, sarcoplasmic reticulum (SR), and mitochondria. Particularly, the SR and or mitochondria play an important role in the fine-tuning of this metabolic process. Recent studies showed that obesity and insulin resistance are associated with interactions between the SR and mitochondrial networks (the dynamic tubular reticulum formed by mitochondria), suggesting that metabolic disorders alter Ca^2+^ handling by these organelles. These interactions are facilitated by specific membrane proteins, including ion channels. This review considers the impact of metabolic disorders, such as obesity and type 2 diabetes, on the regulation of [Ca^2+^]_i_ in skeletal muscle. It also discusses the mechanisms by which this occurs, focusing chiefly on the SR and mitochondria networks. A deeper understanding of the effect of metabolic disorders on calcium handling might be useful for therapeutic strategies.

## Introduction

Obesity due to overeating and lack of exercise has become one of the major burdens of modern societies and is associated with many comorbidities, including type 2 diabetes mellitus (T2DM). Skeletal muscle is important for maintaining healthy body composition, physical function, and locomotion. Obesity is likely to cause a decrease in muscle mass and to lower muscle strength, which is associated with decreased mobility (Freedman et al., 2002). Previous studies have shown decreased muscle strength is observed in obese and T2DM patients (Park et al., 2006; Maffiuletti et al., 2007). Consistent with these findings, recent studies using animal models confirmed that obesity and diabetes lead to decreased muscle contractile force normalized to muscle mass and decrease in muscle performance (Eshima et al., 2017b; Hurst et al., 2019). However, an understanding of the mechanism for dysfunction of muscle contraction in obesity and T2DM has not been fully elucidated.

**Figure 1:**
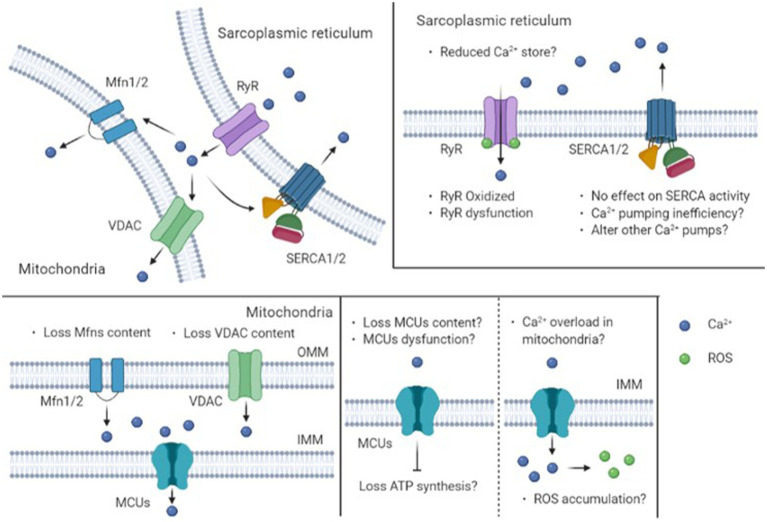
Schematic diagram of intracellular mechanisms for Ca^2+^ regulation by sarcoplasmic reticulum and mitochondria in obese and type 2 diabetic skeletal muscle. Obesity and type 2 diabetes mellitus may cause impairments in Ca^2+^ release capacity *via* a decrease in content or oxidation of RyR. On the other hand, there is no effect on SERCA activity. Note that the mitochondria also have an important sequestration role during the recovery process through a uniporter in OMM or IMM. IMM, The Inner Mitochondrial Membrane; MCUs, Mitochondrial Calcium Uniporter(s); Mfns, Mito Fusion 1 and/or 2; OMM, The Outer Mitochondrial Membrane; ROS, Reactive Oxygen Species; RyR, Ryanodine Receptor; SERCA, Sarcoplasmic reticulum Ca^2+^-ATPase; VDAC, Voltage Gated-Dependent Anion Channel.

Transient elevation of intracellular Ca^2+^ concentration ([Ca^2+^]_i_) is necessary for the initiation of tension development in skeletal muscle tissue (Allen et al., 2008). Dysfunction of muscle contraction in metabolic disease may depend on the impaired capacity for Ca^2+^ release and reuptake by the sarcoplasmic reticulum (SR; Westerblad et al., 2010). These features include decreased SR calcium release and or decreased SR calcium reuptake (Bruton et al., 2002; Bayley et al., 2016). We recently demonstrated Ca^2+^ regulatory impairments during muscle contraction in metabolic disease using the *db/db* mice, a common model of obesity associated with T2DM (Eshima et al., 2019). Similar findings were seen in diet-induced obese mice (Eshima et al., 2020). On the other hand, type 1 diabetes mellitus (T1DM) affects [Ca^2+^]_i_ independent of SR (Eshima et al., 2013, 2015). In this regard, recent studies suggest that mitochondria play a major role in the [Ca^2+^]_i_ buffering with evidence for increased mitochondrial Ca^2+^ concentration ([Ca^2+^]_mito_) during contractions in skeletal muscle (Shkryl and Shirokova, 2006; Ainbinder et al., 2015; Eshima et al., 2017a). Previous studies have shown the [Ca^2+^]_mito_ increases during electrical stimulation-induced contractions in skeletal muscle *in vitro*, suggesting that mitochondria are involved in the regulation of [Ca^2+^]_i_ (Aydin et al., 2009; Rossi et al., 2011; Yamada et al., 2012). Consistent with this, mitochondrial Ca^2+^ uptake is a key supporter of excitation-contraction coupling in skeletal myotubes (Eisner et al., 2014). Indeed, altered [Ca^2+^]_mito_ is a common characteristic of some skeletal muscle myopathies (Debattisti et al., 2019; Favaro et al., 2019), suggesting that diabetic myopathy may also display elevated [Ca^2+^]_mito_. This review considers the impact of obesity and diabetes on calcium handling by skeletal muscle, focusing on the SR and mitochondria. We propose that interactions between these organelles in skeletal muscles of obese and T2DM animals and patients alter calcium handling by skeletal muscles (see Figure 1).

## Obesity And Type 2 Diabetes Alter Ca^2+^ Handling In Skeletal Muscle

Ca^2+^ is a ubiquitous intracellular messenger that can regulate different cellular processes in living tissue (Berridge et al., 2003). In particular, it is well known that elevated [Ca^2+^]_i_ and subsequent Ca^2+^ signaling directly regulate cellular metabolism in various tissues. By contrast, obesity has been shown to impair Ca^2+^ homeostasis in adipose, cardiac, B-cell, and liver (Dong et al., 2006; Park et al., 2010; Tong et al., 2016; Wright et al., 2017). Therefore, certain aspects of Ca^2+^ homeostasis in skeletal muscle may also be compromised in obesity and T2DM. Previous findings related to alteration in SR or mitochondrial Ca^2+^ handling by obesity and type 2 diabetes are summarized in Table 1. Bruton et al. demonstrated that *ob/ob* mice (genetic-induced obesity model) impaired Ca^2+^ handling in skeletal muscle fibers (Bruton et al., 2002). Recently, we demonstrated electrically stimulated Ca^2+^ peak levels were reduced by HFD feeding. Indeed, Ca^2+^ peak levels during the ryanodine receptor (RyR) agonist stimulation decrease in this model (Eshima et al., 2020). Consistent with this, *db/db* mice (an obese type 2 diabetes model) displayed impaired contractile force and reduced SR Ca^2+^-ATPase (SERCA) pump expression (Bayley et al., 2016). Similarly, our study demonstrated a substantial degree of impairment in [Ca^2+^]_i_ homeostasis in skeletal muscle of *db/db* mice (Eshima et al., 2019). Muscle contractile force and [Ca^2+^]_i_ levels were both lower during electrical stimulation in this model, suggesting that decreased Ca^2+^ release may contribute to skeletal muscle contractile dysfunction in obese type 2 diabetic rodent models. In addition, Ca^2+^ release induced by the caffeine was decreased in *db/db* mice. Interestingly, dysfunction of Ca^2+^ release and contractile force was improved by endurance of exercise training in *db/db* mice. This study also found a consistent reduction in sarcalumenin, which is a Ca^2+^-binding protein localized in the SR of the intracellular Ca^2+^ store in the skeletal muscles of these mice. This protein decreases in aging animals (O'connell et al., 2008; Andersson et al., 2011). Collectively, it is expected that exercise intervention may increase the Ca^2+^ store content and improve calcium handling and contractile dysfunction associated with diabetic myopathy (Ferreira et al., 2010a).

**Table 1:** Intracellular calcium handling alterations in obese and type 2 diabetic skeletal muscle.

Model	Methods	Assessment	Change	References
*Human*				
T2DM	• Proteomes data analysis • Transcriptome analysis	• SERCAs, RyR1 • MCUR1, VDACs • Several Ca^2+^ related gene	↑ ↓ ↑	Chae et al., 2018 Gancheva et al., 2019
Obesity and T2DM	• Proteomes data analysis • WB • RT-cPCR • *In situ* proximity ligation assay	• SERCAs • VDAC • Mfn2 • MAM integrity	→ ↓ ↓ ↓	Hwang et al., 2010 Moller et al., 2017 Bach et al., 2003 Tubbs et al., 2018
Severe Obesity	• RNA sequencing and WB • WB • RT-cPCR	• SERCAs, RyR1, SLN • SERCA2 • Mfns • Mfn2	↑ → → ↓	Paran et al., 2015 Kugler et al., 2020 Bach et al., 2005
*Rodent*				
Obese Zucker rats	• Absorption spectrophotometer • WB • Northern blotting and WB	• Intracellular Ca^2+^ content • SERCA1a • Mfn2	↑ or → → ↓	Zemel et al., 1990; Agil et al., 2015 Jain et al., 2014 Bach et al., 2003
HFD rats	• SR fractions • WB • WB	• SERCA activity • S-nitrosylation of the RyR • Mfn2	→ ↑ ↓ or →	Fajardo et al., 2017 Jain et al., 2014 Kong et al., 2013; Leduc-Gaudet et al., 2018
*ob/ob* mice	• [Ca^2+^] flux • Chemical quench technique • WB • *In situ* proximity ligation assay	• [Ca^2+^] transient • Calcium uptake rate • Mfns • MAM integrity	↓ → → ↓	Bruton et al., 2002 Fraser and Trayhurn, 1983 Jheng et al., 2012 Tubbs et al., 2018
*db/db* mice	• Ca^2+^ steady-state rate • WB	• SERCA activity • RyR • SERCAs, DHPR, CSQ	↓ ↓ →	Bayley et al., 2016 Eshima et al., 2020
HFD mice	• [Ca^2+^] flux • WB • WB • WB • WB • SR fractions • WB • Isolated mitochondria • *In situ* proximity ligation assay	• [Ca^2+^] transient • SERCAs and SLN • SERCAs and SLN • SERCAs and CSQ • RyR and DHPR • Phospholamban, SLN • SERCA activity • Mfns • Calcium retention capacity • MAM integrity	↓ → → ↓ → → → ↓ ↓ ↓ ↓ ↓	Eshima et al., 2020 Jaque-Fernandez et al., 2020 Bal et al., 2012 Ciapaite et al., 2015 Eshima et al., 2017a Gamu et al., 2019 Funai et al., 2016 Jheng et al., 2012; Liu et al., 2014; Li et al., 2018 Taddeo et al., 2014 Tubbs et al., 2018

*CSQ, Calsequestrin; DHPR, Dihydropyridine Receptor; HFD, High-Fat Diet; RyR, Ryanodine receptor; SR, Sarcoplasmic reticulum; SERCA, SR Ca^2+^-ATPase; T2DM, Type 2 diabetes mellitus; SLN, Sarcolipin; MAM, Mitochondria-Associated Endoplasmic Reticulum Membrane; MCU, Mitochondrial Calcium uniporter; Mfn, Mito Fusin; VDAC, Voltage Gated-Dependent Anion Channel; WB, Western blotting; RT-cPCR, Reverse Transcription and Competitive Polymerase Chain Reaction*.

## Impact of Obesity and Type 2 Diabetes on Sarcoplasmic Reticulum and Mitochondrial Reticulum

### Sarcoplasmic Reticulum

### Ryanodine Receptor

In skeletal muscle, the increases in [Ca^2+^]_i_ are initiated by sarcolemmal and transverse tubule depolarization, triggering Ca^2+^ release from the SR *via* the RyR. Previous studies demonstrated that T1DM impaired Ca^2+^ release from the SR and decreased RyR protein contents (Eshima et al., 2013, 2015). Similar to T1DM, RyR protein content was decreased in *db/db* mice (Eshima et al., 2019). By contrast, no differences in the RyR protein content between HFD-induced obese mice and mice fed a normal diet (Eshima et al., 2017b; Gamu et al., 2019). On the other hand, oxidation of RyR has been implicated in Ca^2+^ leakage from the SR that causes muscle weakness in aged mice (Andersson et al., 2011). Consistent with this finding, Holloway and colleagues demonstrated that the nitrosylated tyrosine residues on RyRs was increased in HFD-induced obese rats, suggesting that Ca^2+^ leakage from RyR is regulated by reactive oxygen species (ROS; Jain et al., 2014). Ceramides, a family of lipid molecules composed of sphingosine and fatty acid, have been implicated in the induction of oxidative stress in skeletal muscle (Nikolova-Karakashian and Reid, 2011). In murine C2C12 myotubes, direct exposure to ceramide increased ROS and exogenous ceramide depressed diaphragm force production in mice. This weakness was prevented by antioxidant N-acetylcysteine treatment (Ferreira et al., 2010b). Interestingly, sphingosine blocks Ca^2+^ release from RyR and reduces the activity of channels reconstituted into planar lipid bilayers (Sabbadini et al., 1999; Sharma et al., 2000). This suggests that lipid mediators may play important roles in calcium kinetics. Indeed, exogenous sphingolipids and accumulation of ceramide in muscle depresses Ca^2+^ sensitivity of the contractile apparatus (Ferreira et al., 2012). HFD feeding increased muscle ceramides and induced glucose intolerance in mice (Frangioudakis et al., 2010). This provides evidence to support the hypothesis that obesity alters lipid species composition, particularly sphingolipids, and causes impairments in Ca^2+^ release capacity *via* RyR dysfunction.

#### SR Ca^2+^-ATPase

Following muscle contraction, elevated [Ca^2+^]_i_ rapidly decreases due to Ca^2+^ taken up by the SR and other intracellular organelles and returns almost immediately to basal resting levels *via* the activity of SR Ca^2+^-ATPase (SERCA). Most of the studies addressing calcium signaling have been focused on this protein which has a significant influence on skeletal muscle metabolism. A previous study demonstrates reduced SERCA content in *db/db* mouse (Bayley et al., 2016). In contrast, previous studies have shown genetic obese rodent models displayed no changes in SERCA protein expression levels independent of diabetes (Jain et al., 2014; Eshima et al., 2019). Indeed, many studies have shown that HFD-induced obese rodents displayed no changes in SERCA activity and protein content (Bal et al., 2012; Fajardo et al., 2017; Eshima et al., 2017b; Gamu et al., 2019). Phospholipid composition plays a major role in determining membrane fluidity, and HFD feeding is known to alter phospholipid species abundance in mice (Montgomery et al., 2017). A reduction in phosphatidylethanolamine (PE) has been associated with decreased SERCA activity (Funai et al., 2016). A recent study demonstrates the absence of phosphatidylethanolamine N-methyltransferase (PEMT) promotes an increase in metabolic rate and protects from diet-induced obesity, potentially due to decreasing SERCA efficiency in skeletal muscle. However, this lack of PE methyltransferase also causes muscle weakness (Verkerke et al., 2019). In humans, recent studies utilizing muscle biopsies obtained from obese and T2DM patients demonstrate SERCA expression levels were increased compared to muscle from healthy participants (Chae et al., 2018; Gancheva et al., 2019). These data suggest certain aspects of the Ca^2+^ uptake into the SR are upregulated in skeletal muscle of patients with obesity and T2DM patients. Therefore, fundamental questions regarding muscle Ca^2+^ buffering associated with obesity remain to be more fully addressed. It may also be insightful to consider relevant organelles other than the SR in order to resolve the mechanistic basis for Ca^2+^ buffering alterations in obese skeletal muscle.

### Mitochondrial Networks

Mitochondria are the organelles responsible for ATP production by oxidative phosphorylation. Mitochondria are in a dynamic state of fusion and division with respect to other mitochondria forming dynamic tubular structures called mitochondrial networks. Mitochondrial fragmentation may be associated with metabolic disorders (Fealy et al., 2021). A previous study showed that mitochondria-associated endoplasmic reticulum membrane (MAM) integrity, VDAC1-inositol 1,4,5-trisphosphate receptor type1 (IP3R1) interactions are decreased in obese and T2DM patients, suggesting that metabolic disorders alter Ca^2+^ handling by these organelles (Tubbs et al., 2018). Indeed, recent evidence suggests that MAMs contribute to obesity and insulin resistance (Townsend et al., 2020). The mitochondrial networks are regulated by molecular structures in the mitochondrial membranes as indicated below.

#### Voltage-Dependent Anion Channel

Voltage-dependent anion channel (VDAC) is expressed on the outer mitochondrial membrane (OMM) and regulates mitochondrial oxidative phosphorylation. A previous study has shown decreased VDAC proteins and other mitochondrial related proteins in T2DM patients (Moller et al., 2017). VDAC overexpression affects the interaction between SR and mitochondria and enhances [Ca^2+^]_mito_ *in vitro* (Rapizzi et al., 2002). These data suggest mitochondrial dysfunction might contribute to the impaired [Ca^2+^]_i_ regulation in obesity and T2DM.

#### Mitofusin

Mitofusin (Mfn) participates in the fusion of the mitochondrial outer membranes of two adjacent mitochondria and may contribute to Ca^2+^ uptake into the mitochondria (Santel and Fuller, 2001). A previous study demonstrated Mfn2 knockdown decreased in [Ca^2+^]_mito_ after muscle contraction in mouse skeletal muscle (Ainbinder et al., 2015). Many studies have shown that Mfn is implicated in obesity and T2DM [see reviews (Zorzano et al., 2009; Fealy et al., 2018)]. Multiple studies have shown that obese rodent models display decreased Mfn2 in skeletal muscle, but there is no direct evidence of Mfn2 involvement in [Ca^2+^]_mito_ regulation (Bach et al., 2003; Kong et al., 2013; Liu et al., 2014; Li et al., 2018). In addition, SR-mitochondria interactions are reduced in obesity and T2DM *in vivo* and *in vitro*. This suggests the metabolic disease may influence the interactions between these organelles (Tubbs et al., 2018). SR-mitochondria interaction is also required for insulin action as seen by decreases in insulin signaling with dysfunction in organelle interaction. Indeed, silencing Mfn2 attenuates increased mitochondrial Ca^2+^ uptake induced by insulin action in skeletal muscle cells (Del Campo et al., 2014). While direct evidence to support such claims is still lacking, these observations support the hypothesis that obesity and T2DM may contribute to dysfunction of [Ca^2+^]_mito_ regulation.

#### Mitochondrial Ca^2+^ Uniporters

The inner mitochondrial membranes of the mitochondria (IMM) contain mitochondrial Ca^2+^ uptake-related proteins, called the mitochondrial Ca^2+^ uniporters (MCU; Baughman et al., 2011). The MCUs are regulated by mitochondrial Ca^2+^ uptake 1 (MICU1), MICU2, and mitochondrial calcium uniporter regulator 1 (MCUR1), which binds Ca^2+^ with high affinity and promotes uptake by mitochondria (Mammucari et al., 2016). Overexpressing MCU increases the mitochondrial size and causes muscle hypertrophy (Barclay et al., 2007; Mammucari et al., 2015). In contrast, other studies have shown the opposite alteration patterns in mitochondrial calcium uniporter related proteins in T2DM and upregulation of other proteins related to Ca^2+^ transporter/homeostasis (Hwang et al., 2010; Chae et al., 2018). In heart muscle from individuals with T1DM, impaired mitochondrial Ca^2+^ uptake is significantly improved by MCU restoration (Suarez et al., 2018). A previous study demonstrated that the mitochondrial calcium retention capacity is reduced in diet-induced obese mice, suggesting that dysregulation of MCU components is associated with insulin resistance (Taddeo et al., 2014). Collectively, this evidence suggests the possibility of a heretofore unappreciated role for [Ca^2+^]_mito_ regulation in the function of obese and diabetic muscle.

## Conclusion

The present review addressed how obesity and T2DM influence Ca^2+^ handling in skeletal muscle. Recent findings from studies in rodents demonstrated that genetic- and diet-induced obesity has detrimental effects on Ca^2+^ handling. As shown in Figure 1, this observation may be associated with impaired SR Ca^2+^ release and mitochondrial Ca^2+^ uptake. These dysfunctions may be explained at least in part by a decrease in RyR content (or oxidation), a decrease in VDAC, mitofusin, and MCU in diabetic and obese skeletal muscle. These mechanisms are likely to be responsible for the muscle weakness that occurs as a result of obesity and T2DM and may prove useful for defining the optimal therapeutic strategies. Previous studies showed that patients with malignant hyperthermia have altered SR Ca^2+^ release and impaired glucose tolerance (Altamirano et al., 2019; Bojko et al., 2021), suggesting that calcium handling is associated with glucose metabolism. Although mitochondrial calcium uniporter influences on systemic metabolism (Gherardi et al., 2019), the effect of mitochondrial Ca^2+^ uptake on metabolic disorders has not been fully clarified. Future studies will allow us to determine potential physiological mechanisms involving the SR and mitochondrial networks that are responsible for the impairment of Ca^2+^ homeostasis in skeletal muscle under conditions of metabolic disease.

## Author Contributions

HE drafted the manuscript, prepared a figure, edited, and revised the manuscript and approved the final version of manuscript.

## Funding

This article was supported by the KAKENHI from the Ministry of Education, Culture, Sports, Science, and Technology of Japan.

## Conflict of Interest

The author declares that the research was conducted in the absence of any commercial or financial relationships that could be construed as a potential conflict of interest.

## Publisher’s Note

All claims expressed in this article are solely those of the authors and do not necessarily represent those of their affiliated organizations, or those of the publisher, the editors and the reviewers. Any product that may be evaluated in this article, or claim that may be made by its manufacturer, is not guaranteed or endorsed by the publisher.

## Abbreviations

[Ca^2+^]_i_: Intracellular Ca^2+^ concentration
[Ca^2+^]_mito_: Mitochondrial Ca^2+^ concentration
CSQ: Calsequestrin
DHPR: Dihydropyridine receptor
HFD: High-fat diet
IMM: Inner mitochondrial membranes of the mitochondria
IP3R1: inositol 1,4,5-trisphosphate receptor type 1
MAM: Mitochondria-associated endoplasmic reticulum membrane
MCU: Mitochondrial Ca^2+^ uniporter
MCUR1: Mitochondrial calcium uniporter regulator 1
Mfn: Mitofusin
MICU1: Mitochondrial Ca^2+^ uptake 1
MICU2: Mitochondrial Ca^2+^ uptake 2
OMM: Outer mitochondrial membrane
PE: Phosphatidylethanolamine
PEMT: phosphatidylethanolamine N-methyltransferase
ROS: Reactive oxygen species
RyR: Ryanodine receptor
SERCA: SR Ca^2+^-ATPase
T1DM: Type 1 diabetes mellitus
T2DM: Type 2 diabetes mellitus
VDAC: Voltage-dependent anion channel
SLN: Sarcolipin
SR: Sarcoplasmic reticulum
